# Oculomotor Adaptation Elicited By Intra-Saccadic Visual Stimulation: Time-Course of Efficient Visual Target Perturbation

**DOI:** 10.3389/fnhum.2016.00091

**Published:** 2016-03-09

**Authors:** Muriel T. N. Panouillères, Valerie Gaveau, Jeremy Debatisse, Patricia Jacquin, Marie LeBlond, Denis Pélisson

**Affiliations:** Integrative Multisensory Perception Action and Cognition Team, Lyon Neuroscience Research Center, INSERM, Unit 1028, CNRS Unit 5292, Lyon I UniversityLyon, France

**Keywords:** eye movements, sensorimotor integration, adaptation, error processing, mislocalization, saccadic suppression

## Abstract

Perception of our visual environment strongly depends on saccadic eye movements, which in turn are calibrated by saccadic adaptation mechanisms elicited by systematic movement errors. Current models of saccadic adaptation assume that visual error signals are acquired only after saccade completion, because the high speed of saccade execution disturbs visual processing (saccadic “suppression” and “mislocalization”). Complementing a previous study from our group, here we report that visual information presented during saccades can drive adaptation mechanisms and we further determine the critical time window of such error processing. In 15 healthy volunteers, shortening adaptation of reactive saccades toward a ±8° visual target was induced by flashing the target for 2 ms less eccentrically than its initial location either near saccade peak velocity (“PV” condition) or peak deceleration (“PD”) or saccade termination (“END”). Results showed that, as compared to the “CONTROL” condition (target flashed at its initial location upon saccade termination), saccade amplitude decreased all throughout the “PD” and “END” conditions, reaching significant levels in the second adaptation and post-adaptation blocks. The results of nine other subjects tested in a saccade lengthening adaptation paradigm with the target flashing near peak deceleration (“PD” and “CONTROL” conditions) revealed no significant change of gain, confirming that saccade shortening adaptation is easier to elicit. Also, together with this last result, the stable gain observed in the “CONTROL” conditions of both experiments suggests that mislocalization of the target flash is not responsible for the saccade shortening adaptation demonstrated in the first group. Altogether, these findings reveal that the visual “suppression” and “mislocalization” phenomena related to saccade execution do not prevent brief visual information delivered “in-flight” from being processed to elicit oculomotor adaptation.

## Introduction

Every day, human beings make about ~100,000 saccadic eye movements to visually explore their environment. These fast and accurate movements of both eyes are essential for fine vision as they allow redirecting the line of gaze toward objects of interest. The accuracy of these movements is maintained despite physiological, pathological or environmental changes thanks to motor adaptation mechanisms. Indeed, when our eyes repeatedly miss their goal, saccades will be progressively adapted to restore movements’ accuracy (for reviews, see Hopp and Fuchs, 2004; Tian et al., 2009; Iwamoto and Kaku, 2010; Pélisson et al., 2010; Prsa and Thier, 2011). Such saccadic adaptation requires the detection of an error signal, carrying the information about saccadic inaccuracy.

An early view posited that this error signal was the post-saccadic visual error, defined as the distance between the saccadic goal and the gaze direction after saccade termination (reviewed in Hopp and Fuchs, 2004; Pélisson et al., 2010). A more recent view is that saccadic adaptation is elicited by the sensory prediction error that derives from the comparison between the experienced post-saccadic visual error defined above and the predicted one (Bahcall and Kowler, 2000; Wong and Shelhamer, 2011, 2012; Collins and Wallman, 2012; Herman et al., 2013). According to this hypothesis, the predicted post-saccadic visual error is computed thanks to the efference copy signal that encodes the saccade displacement. Both views assume that the experienced visual error is sampled only after the saccade termination. This assumption logically arises from the saccadic suppression phenomenon, which corresponds to an elevation of the threshold to detect visual information and visual displacement during saccade execution (Zuber and Stark, 1966; Matin, 1974; Bridgeman et al., 1994). Indeed, in the target double-step paradigm (to be detailed below) used in nearly all saccadic adaptation studies, saccadic suppression is believed to mask the target jump elicited during the saccade, and thus to delay the processing of the corresponding visual error information until after the saccade.

However on theoretical grounds, given that saccadic suppression contributes to the maintenance of cognitive perceptual stability despite eye movements by preventing signals of an unstable world from reaching awareness, saccadic suppression should *a priori* not prevent the processing of all visual input during saccades. For example, signals which may be desirable for controlling fast and automatic motor responses should escape saccadic suppression. This definition of saccadic suppression is consistent with the theoretical framework of partly separate visual systems in charge of conscious visual perception and of automatic visuo-motor control (Goodale et al., 1986; Milner and Goodale, 1993; Goodale and Westwood, 2004). In fact, it has been demonstrated that the location of a brief visual target presented during the saccade execution phase can be processed for the control of subsequent oculomotor (Hallett and Lightstone, 1976b; Prablanc et al., 1978; Eggert et al., 1999) and limb motor responses (Cameron et al., 2009) and even, under particular conditions, for the on-line control of the same saccade (Zee et al., 1976; MacAskill et al., 2000; Gaveau et al., 2003).

In the laboratory, saccadic adaptation is induced via error signals generated by the double-step target paradigm (McLaughlin, 1967). This paradigm allows creating an artificial saccadic inaccuracy by jumping the saccadic target to a new location at the onset of the saccade, leading the eyes to miss their goal when the saccade ends. When this type of trials is repeated, a progressive change of saccadic amplitude is observed, so that the eyes land closer to the jumped target. Adaptive saccade shortening or lengthening can be achieved by systematically jumping the target backward or forward (against or along saccade direction, respectively). The double-step target paradigm is usually used in a way that the jumped target remains illuminated for a few hundred of milliseconds after saccade termination, providing ample time for visual error processing both during and after the saccade execution. By presenting the jumped target both during and after saccade completion, this approach has thus not been able to evaluate the hypothesis that the experienced visual error is only sampled after the saccade termination. To this end, one needs to directly test whether a brief visual perturbation during the intra-saccadic phase could be sufficient to elicit saccade adaptation. As a first attempt to address this question, Panouillères et al. (2013) modified the double-step target paradigm by shortening the duration of the jumped target such as to suppress any processing of post-saccadic visual information. They revealed that a significant saccade adaptation could be elicited with this “intra-saccadic” visual error presented for the duration of the saccade (~30 ms). Then, by further reducing the intra-saccadic error duration to only 10 ms, they disclosed that adaptation occurred when the visual error is presented during the saccade deceleration phase, but not during the acceleration phase. Strongly suggesting that intra-saccadic target information is an adequate error signal for saccade adaptation, this study also raised the following questions about the implied intra-saccadic processing. First, what are the temporal dynamics of such visual processing during saccade execution? Second, is this intra-saccadic processing related to target mislocalization, a sensory phenomenon that has been long known to occur when the target is briefly flashed during saccade execution (Matin and Pearce, 1965; Honda, 1989, 1990, 1991; Dassonville et al., 1992)? Third, is it capable of inducing an adaptive saccadic shortening and an adaptive saccadic lengthening similarly, given that these adaptive responses rely on partially different processes (Catz et al., 2008; Ethier et al., 2008; Golla et al., 2008; Hernandez et al., 2008; Panouillères et al., 2009, 2012b, 2015; Zimmermann and Lappe, 2010; Schnier and Lappe, 2011, 2012)?

The present study was aimed at addressing these questions. We modified the double-step target paradigm to present the jumped target for only 2 ms. In experiment I, this target flash occurred near the time of saccade peak velocity, of peak deceleration or of saccade termination. In this experiment, the target was jumped backward to induce an adaptive shortening of saccade. In experiment II, we investigated whether the most efficient intra-saccadic timing of experiment I could also allow an adaptive lengthening of saccades. To do so, the 2 ms target flash jumped forward near the time of peak deceleration. Finally, “CONTROL” conditions consisted of presenting the 2 ms flash at the target initial position either near saccade termination (Experiment I) or peak deceleration (Experiment II).

## Materials and Methods

### Subjects

Twenty-four healthy subjects participated in the study: 15 subjects were enrolled in the first experiment (10 Females, mean age = 23 ± 4 years old) and nine subjects in the second experiment (5 Females, mean age = 27 ± 10 years old). Participants were naïve to the goals of the study, except one author in each group. All subjects had normal or corrected-to-normal vision, and no history of neurological or psychological disorder. Subjects gave their informed written consent and were financially compensated for their participation. All procedures complied with the Ethical Principles of the World Medical Association (Declaration of Helsinki) and were approved by the local ethics committee of the Lyon Neuroscience Research Center.

### Apparatus

The apparatus is presented in details in our previous article (Panouillères et al., 2013). Experiments took place in a completely dark room. Subjects were seated 114 cm away from a concave spherical board with their head held still using forehead and chin rests. Red light-emitting diodes (LEDs, diameter: 3 mm; luminance: 12 cd/m^2^; wavelength: 625 nm) located along the horizontal meridian of the board were used as central fixation point (at 0°) and primary (+8° or −8°) or secondary visual targets (+11.2°, +10°, +6°, +4.8° or −11.2°, −10°, −6°, −4.8°). The horizontal position of the right eye was recorded at 1000 Hz with the Eyelink 1000 eye tracker (tower mount set-up, SR Research, Canada). Before each recording session, the eye tracker was calibrated by asking the subjects to successively fixate three LEDs: one located in the central position (0°) and two presented at ±12°. Custom real-time software was used for the on-line monitoring of eye movements, the recording of eye movements for off-line analysis (1000 Hz sampling frequency) and the control of visual stimuli based on the instantaneous eye velocity or acceleration signals (calculated on-line by applying to the raw eye position signal a running difference algorithm with nine points and four points for the 1st and 2nd derivations, respectively).

### Procedure

In both experiments, the secondary target was flashed for 2 ms at different locations relative to the primary target and at different times during the saccade trajectory. Experiment I focused on the saccade shortening adaptation: the secondary target flash was thus presented at a less eccentric location than the primary target and occurred, in three separate conditions, either near the time of peak velocity (“PV”), of peak deceleration (“PD”) or of saccade end (“END”). In the fourth condition (“CONTROL”), the flash occurred at saccade end and at the same location as the primary target (no target step). Experiment II tested whether a saccade lengthening adaptation could be elicited by a target flashed during saccade deceleration: the secondary target flash occurred at the time of peak deceleration, either at a more eccentric location than the primary target or at the same location (in the “PD” and “CONTROL” conditions, respectively). All 15 and 9 subjects of the two groups performed respectively the four conditions of experiment I and the two conditions of experiment II. Each condition was performed in separate sessions taking place on different days, with a minimum delay of 5 days in-between sessions. The order of the conditions was randomly counterbalanced between subjects in each experiment. Each condition comprised exposure blocks of trials with a secondary target flash (adaptation or control) and pre- and post-exposure blocks where only the primary target was presented.

### Pre- and Post-Exposure Blocks

In every condition of the two experiments, identical pre- and post-exposure blocks were performed immediately before and after the two exposure blocks. Each trial lasted from 2000 to 3000 ms and started with the central fixation point (0°) presented for a random duration comprised between 500 and 1500 ms, after which the peripheral target appeared at −8° or +8°. Upon initiation of the saccade toward the target (detected on-line based on a 30°/s velocity threshold), the target was turned off and the subjects had to complete the saccade in darkness, and about a second later (while still in darkness) they had to look back to the center of the screen in preparation for the next trial (continuous monitoring allowed us to remind the subject, if necessary, about the correct timing of his/her look-back saccade). Each pre- and post-exposure block consisted of 12 rightward and 12 leftward trials presented in a random order.

### Exposure Blocks

Each exposure comprised 2 blocks of 96 trials (expo1 and expo2) lasting from 2000 to 3000 ms each. Each trial started like the pre- and post-exposure trials: a central fixation point was presented and then replaced by a peripheral visual target located randomly at −8° or +8°, and finally the detection of the target-directed saccade (30°/s velocity threshold) triggered the extinction of the target. From that point, exposure trials differed depending on the experimental conditions. In experiment I, the secondary target (stepped target) flashed for 2 ms near the time of saccade peak velocity, peak deceleration or termination, respectively, for the “PV”, “PD”, and “END” conditions. To achieve these three different conditions, the target flash was actually triggered 10 ms after eye velocity exceeded the 30°/s threshold, 10 ms after the first time the absolute eye acceleration dropped below 5000°/s^2^, and immediately when eye velocity fell below 30°/s, respectively. The exact time of occurrence of the flash during the saccade is reported in the “Results” section. In the “PV”, “PD”, and “END” conditions, the flash was presented at ±6° (a 25% “backward jump” relative to the primary target) for the first block of trials (expo1: 48 trials in each direction) and at ±4.8° (a 40% “backward jump” relative to the primary target) for the second block (expo2: again 48 trials in each direction). In the “CONTROL” condition of experiment I, the triggering of the flash was similar to the one in the “END” condition, but the flash was presented at the primary target location (±8°). In experiment II, the target flash occurred during the saccade deceleration phase (same timing as in the “PD” condition of experiment I), either at the same location as the primary target for the “CONTROL” condition (±8°) or at a larger eccentricity in the first and second block of the “PD” condition (±10° or ±11.2°, corresponding to a 25% or 40% “forward jump”). Exposure blocks of Experiment II were otherwise identical to those of Experiment I.

### Off-Line Data Analysis

Eye movement data were analyzed off-line using laboratory made software developed with Matlab v.7.10 (Mathworks, MA, USA). The position and time of the beginning and end of each primary saccade were detected using a velocity threshold of 50°/s. The following parameters were calculated: horizontal saccade duration (difference between offset time and onset time of saccade), peak velocity (maximum velocity), saccade amplitude (difference between final and initial eye positions measured 50 ms after saccade termination and 50 ms before saccade onset, respectively), retinal eccentricity (difference between target position and saccade starting position) and saccadic gain (ratio between horizontal saccade amplitude and retinal eccentricity). The mean gain, peak velocity and duration of saccades were calculated, separately for the different conditions, for rightward and leftward saccades, in the pre- and post-exposure blocks as well as in the two exposure blocks (expo1 and expo2). Saccades contaminated with a blink, not correctly detected on-line or with a gain outside [mean ± 3 SD] were excluded from further analysis (this last criteria was applied at the individual level, for each saccade direction and each block separately). The gain change of each saccade recorded during the exposure and post-exposure blocks was calculated with respect to the mean gain of the corresponding pre-exposure block, separately for rightward and leftward saccades. Positive gain changes represent a modification of saccade amplitude in the expected direction relative to the adaptive training (decrease and increase in Experiments I and II, respectively), whereas negative values represent gain changes in the opposite unadapted direction. The timing of the target flash was determined for each saccade by measuring the delay between the onset of the stored LEDs pulse signal and either the time of peak velocity (“PV-Flash” delay) or the time of saccade offset (“Flash-End” delay). The position of the eye at the time of target flash was then measured for each exposure trial of Experiment I.

Statistical analyses were performed using Statistica 9 (Statsoft Inc., Tulsa, OK, USA). Saccade gain was submitted to three-way ANOVAs with the following factors: Condition (experiment I: “PV”, “PD”, “END” and “CONTROL”; experiment II: “PD”, “CONTROL”), Block of trials (pre-, expo1, expo2, post-) and Target side (left, right). In Experiment I, follow-up analyses of saccade kinematics were performed by applying the same three-way analysis of variances (ANOVAs) to saccade peak velocity and duration. Significant ANOVAs were followed by *post hoc* comparisons (HSD Tukey test). Significant threshold was set at *p* < 0.05.

## Results

### Experiment I

**Figure 1:**
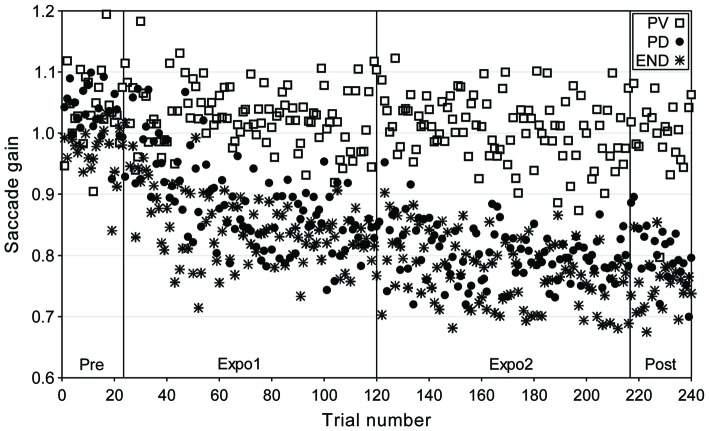
**Time-course of gain changes for a representative subject (BL) in the “PV”, “PD” and “END” conditions of experiment I (adaptive saccade shortening).** Saccadic gain is plotted as a function of trial number for the different experimental phases: Pre (trials 1–24); exposure 1 (Expo1: trials 25–120); exposure 2 (Expo2: trials 121–216) and Post (trials 217–240). Different symbols represent the gain of saccades in the “PV” (open squares), “PD” (filled circles) and “END” (star) conditions.

We plot in Figure 1 the data of one representative subject for the three main experimental conditions (“CONTROL” condition not shown) where the target was flashed backward for 2 ms near the time of peak velocity (“PV”), of peak deceleration (“PD”) and of saccade termination (“END”). The saccade gain appears to only slightly decrease over the duration of the “PV” condition while it showed a strong and consistent reduction both in the “PD” and “END” conditions. This gain decrease persisted in the post-exposure block (trials 217–240) where the target simply disappeared at saccade detection, suggesting that this decrease resulted from an adaptive process and not a strategic response.

**Figure 2:**
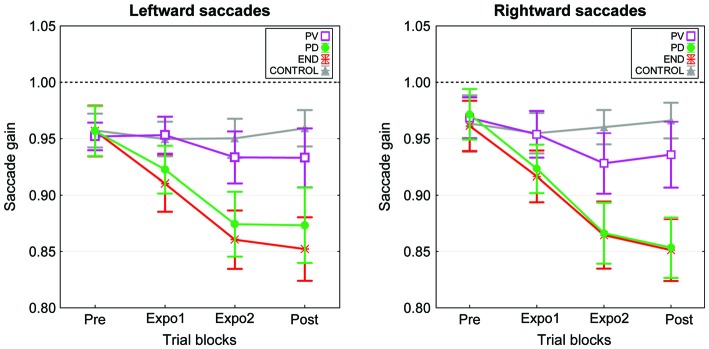
**Time-course of the mean gain changes in the four experimental conditions of experiment I.** The mean saccade gain calculated across the 15 subjects is plotted as a function of the experimental blocks (Pre, Expo1, Expo2 and Post) separately for the leftward saccades (left panel) and the rightward saccades (right panel). The “PV” (purple), “PD” (green), “END” (red) and “CONTROL” (gray) conditions are represented superimposed. Error bars are SEMs.

We then computed for each subject the mean gain separately for the four blocks (pre, expo1, expo2 and post) of each condition. Plots in Figure 2 represent the grand mean of saccade gain averaged over the 15 subjects. Note first that for each saccade direction (target at −8° or 8°), the change in gain was not identical for the four experimental conditions. Indeed the gain decrease was substantial in both the “PD” and “END” conditions while it was either very small or absent respectively in the “PV” and “CONTROL” conditions. This observation is confirmed by the results of a three-way repeated-measures ANOVA testing the effects of the factors Condition, Block and Target on saccadic gain. This ANOVA disclosed a significant effect of Condition (*F*_(3,42)_ = 4.27, *p* = 0.01), Block (*F*_(3,42)_ = 18.2, *p* < 0.001), and a significant interaction between Condition and Block (*F*_(9,126)_ = 9.2, *p* < 0.001). This interaction arises because, on one hand, the gain in all four blocks was similar between the “PD” and “END” conditions (*post hoc* Tukey test: *p* > 0.05) as well as between the “PV” and “CONTROL” conditions (*p* > 0.05) and on the other hand, the saccade gains in the “PD” and “END” conditions significantly differed from those of the “PV” and “CONTROL” conditions in expo2 and post-exposure blocks (*post hoc* Tukey: *p* < 0.05, a significant difference is further revealed between “PV” and “END” in expo1 block).

To summarize, when compared to the “CONTROL” condition, saccade gain in “PD” and “END” conditions decreased, reaching a significant level in the second exposure block and in the post block. In addition, there was no difference of gain between the “PD” and “END” conditions. However, the gain in the “PV” condition did not significantly differ from the one of the “CONTROL”. These findings demonstrate that a strong and significant saccade shortening adaptation could be elicited using as the error signal a target flashed for 2 ms during the saccade deceleration phase or near the saccade termination.

**Figure 3:**
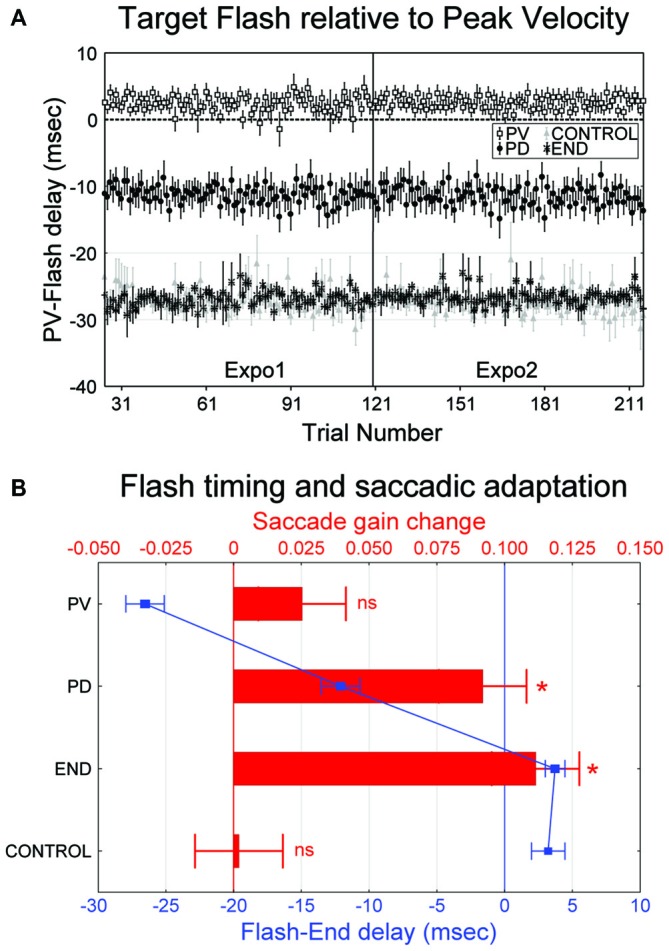
**(A,B)** Occurrence of the 2 ms flash and its relation with saccadic gain change (experiment I). **(A)** The mean onset time of the target flash relative to the time of peak velocity (“Flash-PV” delay) across the 15 subjects is plotted as a function of trial number. Negative (positive) values indicate flash following (preceding) the saccade peak velocity. Error bars are SEMs. **(B)** Mean change of saccadic gain in post relative to pre (red bars, upper *x*-axis) and mean onset time of the flash relative to saccade end (“Flash-END” delay, blue curves and symbols, lower *x*-axis) are represented as a function of the four experimental conditions (“PV”, “PD”, “END” and “CONTROL”). Positive gain changes indicate gain decrease. Significant differences of gain changes relative to zero (Student *t*-test) are indicated by: *p < 0.05. Negative values of “Flash-END” delay indicate intra-saccadic flash. Errors bars are SEMs.

The actual timing of the target flash relative to the time of saccade peak velocity (“PV-Flash” delay, see “Materials and Methods” Section) is shown in Figure 3A for the four experimental conditions. Despite some trial-to-trial fluctuation, the flash timing remained remarkably stable throughout the exposure blocks. Note that, in the “PV” condition, the flash occurred slightly before the actual time of peak velocity (2.4 ± 1.8 ms on average). Moreover, the flash timing in the “PD” condition (−11.4 ± 1.4 ms) was intermediate between the “PV” condition on one hand, and the “END” and “CTRL” conditions on the other hand (−26.9 ± 1.9 and −27.2 ± 1.6, respectively).

In order to more directly determine the temporal dynamics of intra-saccadic visual processing for saccadic adaptation and to clarify to what extent the target flash occurred exclusively during the intra-saccadic period or not, we re-plot in Figure 3B the timing of the visual flash, this time measured relative to saccade offset. Across the four conditions, this “Flash-End” delay (see “Materials and Methods” Section) can be associated with its amount of induced adaptation (post- vs. pre-gain change). Due to technical lag between on-line eye signals processing and target triggering, flashes in the “END” and “CONTROL” conditions occurred actually 3–4 ms after the termination of saccades measured off-line, whereas in the “PV” and “PD” conditions, they preceded saccade termination by approximately 27 and 12 ms, respectively. Concerning the resulting saccade gain changes, a significant decrease of gain occurred for both “PD” (0.092 ± 0.113) and “END” conditions (0.111 ± 0.088, one sample *t*-test comparison to zero: *t*_(29)_ = 4.47, *p* < 0.001 and *t*_(29)_ = 6.96, *p* < 0.001, respectively). However, no significant gain change was detected for “PV” (0.025 ± 0.089) or “CONTROL” (0.002 ± 0.056, one sample *t*-test comparison to zero: *t*_(29)_ = 1.55, *p* > 0.05 and *t*_(29)_ = 0.19, *p* > 0.05, respectively). When expressed as relative changes, the gain was reduced in post-relative to pre- by 0.3 ± 5%, 3.8 ± 11%, 12.8 ± 13% and 14.1 ± 10% in the “CONTROL”, “PV”, “PD” and “END” conditions, respectively.

We next measured the saccade kinematics in the different phases of the four conditions. Table 1 summarizes the mean values of saccade peak velocity and duration. A three-way repeated measures ANOVA (factors: Condition, Block and Target side) disclosed a significant Condition × Block interaction for both peak velocity and duration (*F*_(9,126)_ = 4.6 and 4.7, respectively, *p* < 0.001). As seen in Table 1, these interactions reflect a decrease of both peak velocity and duration in the “PD” and “END” conditions with respect to the “CONTROL” and “PV” conditions where a much smaller decrease of peak velocity or a slight increase of duration are observed. These changes are thus consistent with the decrease in gain, reported above, in “PD” and “END” conditions relative to the “CONTROL” and “PV” conditions.

**Table 1:** **Saccade kinematic parameters (Experiment I)**.

Condition	Block	Duration (ms)	Peak vel (°/s)	Eye @target flash (°)
CONTROL	Pre	42.9 (3.9)	295 (42)	–
	Expo1	43.2 (3.6)	292 (46)	7.6 (0.5)
	Expo2	43.4 (4.0)	294 (46)	7.6 (0.5)
	Post	43, 8 (3.5)	294 (46)	–
PV	Pre	41.9 (0.7)	303 (46)	–
	Expo1	42.6 (3.8)	300 (51)	2.8 (0.8)
	Expo2	43.1 (4.0)	291 (51)	2.7 (0.8)
	Post	42.7 (3.8)	295 (54)	–
PD	Pre	42.4 (4.6)	296 (45)	–
	Expo1	42.2 (4.5)	287 (45)	6.0 (1.5)
	Expo2	41.8 (4.7)	275 (47)	5.9 (1.7)
	Post	42.0 (4.4)	273 (46)	–
END	Pre	42.4 (3.6)	294 (43)	–
	Expo1	41.9 (3.9)	288 (42)	7.3 (0.8)
	Expo2	41.0 (3.8)	278 (45)	6.9 (0.8)
	Post	41.0 (3.6)	274 (44)	–

*Mean (SD) values of duration, peak velocity and eye position at time of target flash are listed for each block and condition*.

We also report in Table 1 (last column) the mean position of the eye measured at the time of target flash. Given these values, and the spatial location of the target flash (6° in expo1 and 4.8° in expo2), we derived the following average retinal location of the flashed target : “PV”= 3.2°, “PD”= 0°, “END”= −1.3° and “CONTROL”= −1.6° for expo1 and “PV”= 2.1°, “PD”= −1.1°, “END”= −2.1° and “CONTROL”= −2.8° for expo2 (positive numbers correspond to target flash ahead of fovea). Thus during expo1 of the “PD” condition, the target was presented on average at 0° relative to the fovea, but further away in the “PV” condition (+3.2° ahead of fovea) and “END” condition (−1.3° past the fovea). So neither the sign nor magnitude of experienced retinal error relative to the fovea seems to explain the differences of adaptation level between the different conditions, consistent with the view that adaptation is rather elicited by a sensory prediction error (see “Introduction” Section).

In sum, a difference of about 15 ms for the timing of the intra-saccadic visual error between the “PV” and “PD” conditions is accompanied by a marked difference of adaptation levels. Conversely, adaptation levels in the “PD” and “END” conditions were similar despite a further 16 ms difference of flash timing. Therefore, the target flash delay in the “PD” condition was the most efficient intra-saccadic timing to elicit saccade shortening adaptation.

### Experiment II

**Figure 4:**
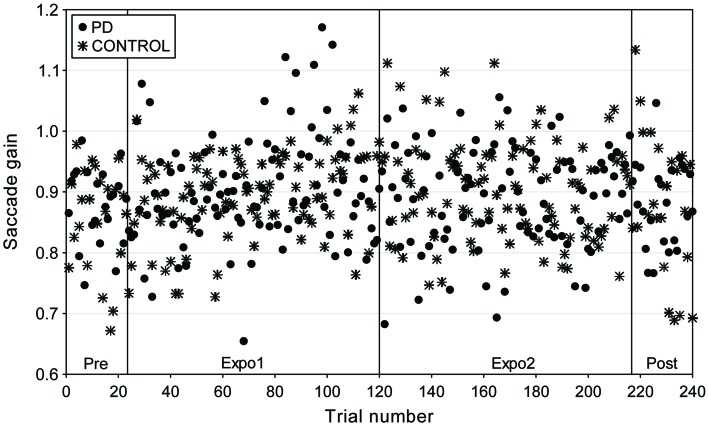
**Time-course of gain changes for a representative subject (PJ) in the “PD” and “CONTROL” conditions of experiment II (adaptive saccade lengthening).** Saccadic gain is plotted as a function of trial number for the different experimental phases: Pre (trials 1–24); exposure 1 (Expo1: trials 25–120); exposure 2 (Expo2: trials 121–216) and post (trials 217–240). Different symbols represent the gain of saccades in the “PD” (filled circles) and “CONTROL” (star) conditions.

Experiment II was designed to test whether a forward jumping target near the time of saccade peak deceleration would lead to a saccade lengthening adaptation. Figure 4 plots the gain of saccades collected in this “PD” condition, superimposed with the data in the “CONTROL” condition (no jump), for a representative subject. Contrarily to experiment I, the time-course of saccade gain overlapped for the two conditions as the saccade gain remained stable for both experimental conditions.

**Figure 5:**
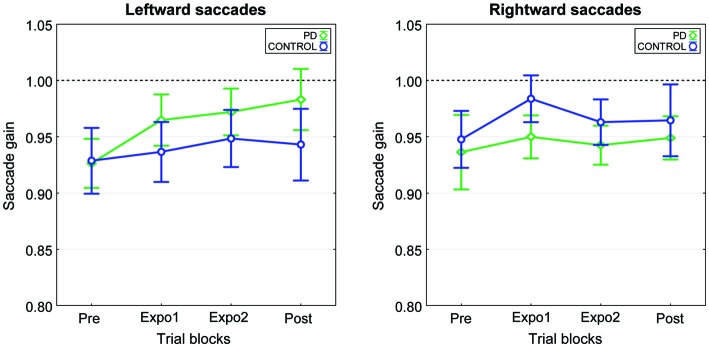
**Time-course of the mean gain changes in the two experimental conditions of experiment II.** The mean saccade gain calculated across the nine subjects is plotted as a function of the experimental blocks (Pre, Expo1, Expo2 and Post) separately for the leftward saccades (left panel) and the rightward saccades (right panel). The “PD” (green) and “CONTROL” (blue) conditions are represented superimposed. Error bars are SEMs.

We computed for each subject the mean gain separately for the four blocks (pre, expo1, expo2 and post) of each condition. Plots in Figure 5 represent the grand mean of saccade gain averaged over the nine subjects. Note first that for each saccade direction (target at −8° or 8°), changes in gain were small and of similar magnitude for the two conditions. This lack of systematic gain change was confirmed by the results of a three-way repeated-measures ANOVA testing the effects of the factors Condition, Block and Target on saccadic gain. Indeed, beside a significant Condition × Target interaction (*F*_(1,8)_ = 6.7, *p* = 0.032) which is related to a slightly larger gain of leftward saccades in the “PD” condition than in the “CONTROL” condition and to an opposite trend for rightward saccades (Figure 5), this analysis disclosed no effect of any main factor nor of any other interaction. In particular, there was no significant effect of the Block factor, showing that the saccadic gain remained constant throughout the experiment. In both conditions, the slight increase of gain in post relative to pre did not significantly differ from zero (overall gain changes: “PD”= 0.035 ± 0.094, “CONTROL”= 0.016 ± 0.092; *t*_(17)_ > 0.72, *p* > 0.05). When expressed as relative changes, the gain was increased in post-relative to pre- by 2.0 ± 8% and 4.4 ± 11% in the “CONTROL” and “PD” conditions, respectively. In this experiment, the timing of the flash target relative to the end of the saccade (“Flash-End” delay, see “Materials and Methods” Section) was by design, very similar between the two conditions (−20.5 ± 2.4 ms in “PD”, −19.2 ± 2.3 ms in “CONTROL”). Note that in experiment II, the “PD” timing occurred a bit earlier than in experiment I (−12.1 ± 1.4 ms), but was still 5.5 ± 1.4 ms after the saccadic peak velocity (“PV-Flash” delay) and so still occurred during the deceleration period. Thus in experiment II, this timing was unable to elicit a significant increase of saccade size.

These observations allow us to conclude that a 2 ms error signal presented near the time of peak deceleration was unable to trigger any significant adaptive lengthening.

## Discussion

In a previous study from our group (Panouillères et al., 2013), we were able to show that a visual error presented solely during saccade execution (not extending to the post-saccade period) could induce both adaptive lengthening and shortening of saccades. We also showed that an error signal presented for only 10 ms during the deceleration phase but not the acceleration phase of the saccade could trigger adaptive saccade shortening. Because these findings contradict the classic view that visual error signals used for adaptation are sampled only after the saccade termination (see “Introduction” Section), we further investigated in the present study the precise time-course of this intra-saccadic visual processing by using an error signal of an even shorter duration (2 ms). We found that adaptive saccade shortening was induced when the error signal (backward target jump) was presented near the time of peak deceleration or saccade termination but not near the time of peak velocity. In contrast, adaptive saccade lengthening was not observed when the forward target jump was presented near the time of peak deceleration. These two main findings are discussed in the following.

The mere presence of a significant adaptation induced by a backward target jump which was flashed for only 2 ms during saccade execution is remarkable and highlights the effectiveness of the visuo-oculomotor system to process intra-saccadic visual error signals for the induction of saccadic adaptation. However no adaptation could be elicited when the same error signal was presented near the time of peak velocity, nor when, as shown previously (Panouillères et al., 2013), a yet longer-lasting signal (10 ms) was presented during the acceleration phase. What could be the reason for this lack of adaptation when the error signal is presented during the initial phase of saccade execution? A first explanation could be the saccadic suppression phenomenon. Indeed, saccadic suppression is known to culminate at the beginning of the saccade, and to decay progressively until after some tens of milliseconds after saccade termination (Bridgeman et al., 1994). However, it has been shown that saccadic suppression is minimal for targets of high luminance (Anand and Bridgeman, 2002) and of high spatial frequencies (Ross et al., 1996) such as the ones used in the present study. In addition, as already mentioned in Introduction, positional information of a brief visual target presented during saccade execution can escape saccadic suppression and be transferred to the motor system. It then seems unlikely that saccadic suppression is responsible for the lack of adaptation observed when the error signal is restricted to the saccade initial phase as in the present “PV” condition. A second explanation is that the eye speed reached during this early saccade phase could be too high to permit a reliable sampling of visual information. For example, Prablanc et al. (1978) have shown that corrective saccades do not reliably follow primary saccades toward a brief visual target when the latter disappears before the saccade mid-deceleration time (eye speed still larger than about 100°/s). We speculate that, at best, some visual processing could take place during the saccade initial phase but would not be accurate enough to be used for motor control. A third and non-exclusive explanation is that the calculation of the error signal for saccadic adaptation may rely on non-retinal signals which are not yet available during this initial phase of saccade execution. According to the dominant hypothesis about the error signal leading to saccadic adaptation (Bahcall and Kowler, 2000; Wong and Shelhamer, 2011, 2012; Collins and Wallman, 2012; Herman et al., 2013), a sensory prediction error is calculated by combining an efference copy signal, encoding the saccade displacement, with the pre-saccadic retinal error, encoding the target distance from the fovea. Consistent with this sensory prediction error hypothesis, the following results of Experiment I indicate that adaptation is not solely related to the experienced target retinal error: (see also Wong and Shelhamer, 2011; Herman et al., 2013): first, a significant shortening adaptation in the “PD” condition despite the target being flashed on average on the fovea and second, the lack of lengthening adaptation in the “PV” condition (an opposite trend being observed) where the target was flashed on average +3.2° ahead of the fovea. We therefore suggest that target error encoding relies on accurate eye position information provided by an efference copy. One possibility is that the efference copy is not yet available until the time of saccade peak velocity. Thus, even if, contrary to the preceding explanation, visual error was adequately processed during the initial saccade phase, the sensory prediction error could not be calculated, and as such, no adaptation could be induced. Our data do not allow disentangling between these two possibilities, but we can clearly state that the early phase of a saccade is not open to error signal processing leading to saccadic adaptation.

From a pure perceptual point of view, it has been shown that targets flashed in the dark shortly before or after as well as during the saccade execution are mislocalized (Matin and Pearce, 1965; Matin et al., 1969; Honda, 1989). Because of the theoretical framework of partly separate visual systems for perception and action (Goodale et al., 1986; Milner and Goodale, 1993; Goodale and Westwood, 2004), there has been a strong interest in investigating whether mislocalization is a pure perceptual phenomenon or whether it also affects motor responses (either eye or arm movements). Several studies have reported accurate motor responses to targets flashed during saccade execution (Hallett and Lightstone, 1976a,b; Bridgeman et al., 1979; Hansen and Skavenski, 1985; Burr et al., 2001; Morrone et al., 2005). However, other studies reported on the contrary that eye and arm movements are not accurately directed toward a target flashed around saccade execution (Honda, 1989, 1990, 1991, 1999; Dassonville et al., 1992, 1995; Miller, 1996; Bockisch and Miller, 1999). Some of these latter studies showed that targets presented at the beginning of the saccade were mislocalized in the saccade direction while targets presented at the end of the saccade were mislocalized in the opposite direction from the saccade (Honda, 1989, 1990, 1991, 1999; Dassonville et al., 1992, 1995). In the current study, we show that the flashed targets had to be presented in the second half of the saccade execution (peak deceleration or saccade end) to induce a significant adaptive decrease. Based on the aforementioned previous works, this target could have been mislocalized in the opposite direction from the saccade. One may then wonder whether the adaptation that occurred in our experiment I could be the result of target mislocalization. Answer to this question comes from our “CONTROL” conditions where the intra-saccadic flash occurred at the target initial position (±8°) at the end or peak deceleration of the saccade (Experiment I or II, respectively). If the mislocalization phenomenon was responsible for saccadic adaptation, then a reduction of saccadic gain should have occurred in these two conditions, which is not the case. This suggests that the adaptive shortening that occurred in experiment I does not result from a mislocalization of the flash by the visual system but rather is a direct consequence of the flashed target being processed as a visual error.

Our results also show that, while an adaptive shortening of saccades was induced when the target was flashed for 2 ms near the time of peak deceleration, the saccadic gain was only reduced by ~13% relative to pre-. This after-effect reveals a lower adaptation relative to that induced in the classical double-step target paradigm where the target is presented both intra- and post-saccadically (e.g., ~17–20% in Panouillères et al., 2011). Moreover intermediate levels of adaptation were found in our previous study (Panouillères et al., 2013) when the visual error was presented for 30 ms (~16–17%) or for 10 ms (~13%). Similarly, Cameron et al. (2009) found that a smaller online hand correction occurred when target information was only presented for 20 ms intra-saccadically than when it was presented until after saccade completion (total duration: 80 ms). Put together, these results suggest that visual information acquired during saccade execution are partly unreliable when target duration is ≤10–20 ms and will then lead to a partial correction of motor responses (either corrective movement or adaptation). Beyond this effect of the flashed target duration on the adaptation strength, both the present study and our previous study (Panouillères et al., 2013) illustrate that intra-saccadic error information is more efficient in inducing an adaptive shortening of saccades than an adaptive lengthening. Indeed, in the present study, the adaptive shortening after-effect reached about 13% (experiment I) whereas the gain increase reached a non-significant level of 4% (experiment II). Similarly, in our previous study using a 30 ms flash, the corresponding values were ~16–17% for the adaptive shortening and 7% for the adaptive lengthening. These observations are in agreement with many previous studies that have shown that adaptive lengthening of saccades is more difficult and slower to develop than adaptive shortening (Miller et al., 1981; Straube and Deubel, 1995; Straube et al., 1997; Noto et al., 1999; Robinson et al., 2003; Alahyane and Pélisson, 2004; Kojima et al., 2004; Panouillères et al., 2009, 2012a,b, 2013, 2015; Zimmermann and Lappe, 2010). We propose here that this well-established difference between the two types of adaptation could be exacerbated when the visual error is provided only during the saccade execution because the size of the retinal error differs between the two conditions. Indeed, in the “PD” condition of the present study, the flashed target actually appeared closer to the fovea in the adaptive shortening paradigm than in the adaptive lengthening (respectively −0.5° as measured in Experiment I and +4.5° as estimated in Experiment II). This is because the eye have traveled about 6° at the time the target flashed in the adaptive shortening (see Table 1) and because the target flashed on average 5° more eccentrically in the adaptive lengthening protocol than in the adaptive shortening. Given that visual processing is more reliable in the center of the visual field, and that small (~2–3°) visual errors elicit optimal adaptation in the monkey (Robinson et al., 2003), we believe that this difference in error size could partly explain the different levels of adaptation between shortening and lengthening when target error signals are provided only intra-saccadically.

The demonstration that saccadic adaptation can be elicited with purely intra-saccadic error signals logically leads to the following questions: are the underlying error processing mechanisms similar to those involved in processing post-saccadic error signals? Do their effects add up when, as in the classical double-step target paradigm, error signals are available both during and after saccadic execution? Regarding the first question, our previous study (Panouillères et al., 2013) disclosed that the same level of adaptation was induced whether the error signal lasting ~30 ms was presented solely during the saccade or after the saccade. A parsimonious explanation of this result is that similar error signal processing mechanisms are involved in both cases. Thus, turning to the second question, one could postulate that their effects add up when the visual error is presented in both intra- and post-saccadic phases, and could then predict a higher adaptation level than when the visual error is presented in either phase alone. However, contrary to this prediction, we previously reported similar adaptation amounts when both intra- and post-saccadic visual errors are available (Panouillères et al., 2011) as when only the post-saccadic visual error is available (Panouillères et al., 2013), every other factors being equal. This observation is thus in line with a hypothesis favoring a non-linear addition between the effects of the “intra-saccadic” and “post-saccadic” error processing mechanisms. This is consistent with the fact that, in many visual areas, post-saccadic response is increased and can mask the intra-saccadic visual information (Ibbotson and Krekelberg, 2011), making it likely that when both intra- and post-saccadic visual errors are presented, the post-saccadic information wins. Accordingly, we postulate that the contribution to saccadic adaptation of intra-saccadic visual information is strong when no visual information is present post-saccadically, as reported in the present and previous (Panouillères et al., 2013) studies, even though it is dominated by post-saccadic visual information when available. Additional studies using conflicting target perturbations during and after saccade execution will be necessary to test this prediction.

In summary, the present study showed that a very brief visual error (2 ms) presented during the saccade deceleration period or at saccade end is sufficient to elicit saccadic adaptation, but this was only true for adaptive shortening but not adaptive lengthening, highlighting again the difference between these two adaptive processes. We demonstrated that this adaption was not a by-product of the mislocalization phenomenon but rather the direct consequence of the target being processed as a visual error. These findings demonstrate the remarkable spatial and time computing capabilities of the visuo-oculomotor system.

## Author Contributions

MTNP: designed the experiment, wrote the article. VG: designed and performed one experiment, analyzed data, corrected article. JD, PJ and MLB: performed experiments and analyzed data. DP: supervised work, analyzed data, wrote article.

## Conflict of Interest Statement

The authors declare that the research was conducted in the absence of any commercial or financial relationships that could be construed as a potential conflict of interest.
